# PRG3 induces Ras-dependent oncogenic cooperation in gliomas

**DOI:** 10.18632/oncotarget.8592

**Published:** 2016-04-05

**Authors:** Zheng Fan, Philipp Bittermann-Rummel, Eduard Yakubov, Daishi Chen, Thomas Broggini, Tina Sehm, Gökce Hatipoglu Majernik, Stefan W. Hock, Marc Schwarz, Tobias Engelhorn, Arnd Doerfler, Michael Buchfelder, Ilker Y. Eyupoglu, Nicolai E. Savaskan

**Affiliations:** ^1^ Translational Neurooncology Laboratory, Department of Neurosurgery, University Hospital Erlangen, Friedrich-Alexander University of Erlangen-Nürnberg (FAU), Erlangen, Germany; ^2^ Department of Neurosurgery, Klinikum Nürnberg, Paracelsus Medical University, Nürnberg, Germany; ^3^ Department of Neurosurgery, Charité - Universitätsmedizin Berlin, Berlin, Germany; ^4^ Department of Neuroradiology, University Hospital Erlangen, Friedrich-Alexander University of Erlangen–Nürnberg, Erlangen, Germany; ^5^ BiMECON ENT., Berlin-Brandenburg, Germany

**Keywords:** glioma, PRG3, Ras, oncogenesis, neuronal plasticity

## Abstract

Malignant gliomas are one of the most devastating cancers in humans. One characteristic hallmark of malignant gliomas is their cellular heterogeneity with frequent genetic lesions and disturbed gene expression levels conferring selective growth advantage. Here, we report on the neuronal-associated growth promoting gene PRG3 executing oncogenic cooperation in gliomas. We have identified perturbed PRG3 levels in human malignant brain tumors displaying either elevated or down-regulated PRG3 levels compared to non-transformed specimens. Further, imbalanced PRG3 levels in gliomas foster Ras-driven oncogenic amplification with increased proliferation and cell migration although angiogenesis was unaffected. Hence, PRG3 interacts with RasGEF1 (RasGRF1/CDC25), undergoes Ras-induced challenges, whereas deletion of the C-terminal domain of PRG3 (PRG3^ΔCT^) inhibits Ras. Moreover PRG3 silencing makes gliomas resistant to Ras inhibition. *In vivo* disequilibrated PRG3 gliomas show aggravated proliferation, invasion, and deteriorate clinical outcome. Thus, our data show that the interference with PRG3 homeostasis amplifies oncogenic properties and foster the malignancy potential in gliomas.

## INTRODUCTION

Malignant gliomas are one of the most common primary brain tumor entities with an annual incidence rate of 5.8 in the USA and in Europe [1, 2]. For malignant glioma patients (WHO grade III and IV including glioblastomas or GBM) a poor prognosis persists despite new generation multimodal therapies and anti-angiogenic approaches [2, 3]. The reason why malignant gliomas resist treatment may reside in their primary characteristics including cellular heterogeneity, rapid proliferation, invasive growth, frequent cell death, and angiogenesis [2, 4]. Recently, progress has been made in the characterization of malignant gliomas revealing various heterogeneous genetic lesions with the potential to transform glial progenitor cells and differentiated glial cells into malignant gliomas (a term indicating their cellular origin) [3, 5–7]. The prevailing concept suggests that tumor-initiating cancer stem cells determine the hierarchical order and complexity of malignant gliomas [8, 9]. However, recent experimental evidences demonstrate that genetic lesions can affect also differentiated neurons and glial cells, thus giving rise to less matured states and subsequently turn into malignant gliomagenesis [10, 11]. Further, gliomas with enriched neural stem cell marker expression display a poor prognosis [10, 11]. It is therefore conceivable that malignant gliomas can develop out of neuronal precursors and differentiated cells with a neuronal gene-profile. In principle, this could explain the expression of neuronal genes often found in malignant gliomas.

In this context, a relatively new group of molecules called Plasticity-Related-Genes (PRGs) appear to play a significant role. To date, 5 members have been characterized (PRG1-5), all belonging to the lipid-phosphate-phosphatase superfamily with primary expression in the central nervous system (CNS) [12–14]. They facilitate axonal growth during embryonic CNS development, induce differentiation, create filopodia and increase regenerative axonal sprouting [15, 16]. PRG3 occupies a special position amongst the PRG family, in that it is expressed more widely in various organs, is regulated during embryonic development with a noticeably higher expression in fetal brains and is characterized by its transformatory activity on cell morphology independent of the cellular origin [13]. PRG3 largely acts as type II transmembrane protein and in the context of neurons impede RhoA dependent axonal collapse signals induced by neurite growth inhibitors such as LPA and Nogo-A [13, 14]. In contrast to other PRGs, PRG3 does not exhibit significant ecto-enzymatic phosphatase activity and in conjunction with this PRG3 does not carry any critical amino-acids within the extracellular domains common in lipid phosphate phosphatases. Moreover, PRG3 has been shown to be expressed in young neurons during development and executes filopodia growth independently of Cdc42 and Rif [13, 17, 18]. The tight regulation of PRG3 during brain development and epileptic seizures indicates the importance in its fine balanced homeostasis during neuronal plasticity [13, 19, 20]. Although the PRG-family is a neuron-associated gene family, PRG3 appears to take up a distinct role with regard to choice of cell populations-it is not restricted to central nervous system tissue but identifiable in other organs-as well as in malignant gliomas.

In this study, we investigated the impact of distinct expression thresholds of PRG3 on glioma morphology and function. In malignant gliomas PRG3 is expressed in opposing amounts in the way that PRG3 is either elevated or down-regulated compared to non-transformed human specimens. Analysis of the human expression databases does at least show that a deregulated expression dosage of PRG3 leads to a worse outcome in patients with malignant gliomas. Deregulated PRG3 expression reduced apoptosis, enhances proliferation, migration and thus elevated the malignancy of glioma cells. Further investigations of the underlying signaling pathway revealed that PRG3 interacts with RasGEF1. Thus, interference with the regulation and homeostasis of PRG3 amplifies malignancy in glioma cells.

## RESULTS

### Disturbed PRG3 expression in human malignant gliomas

**Figure 1:**
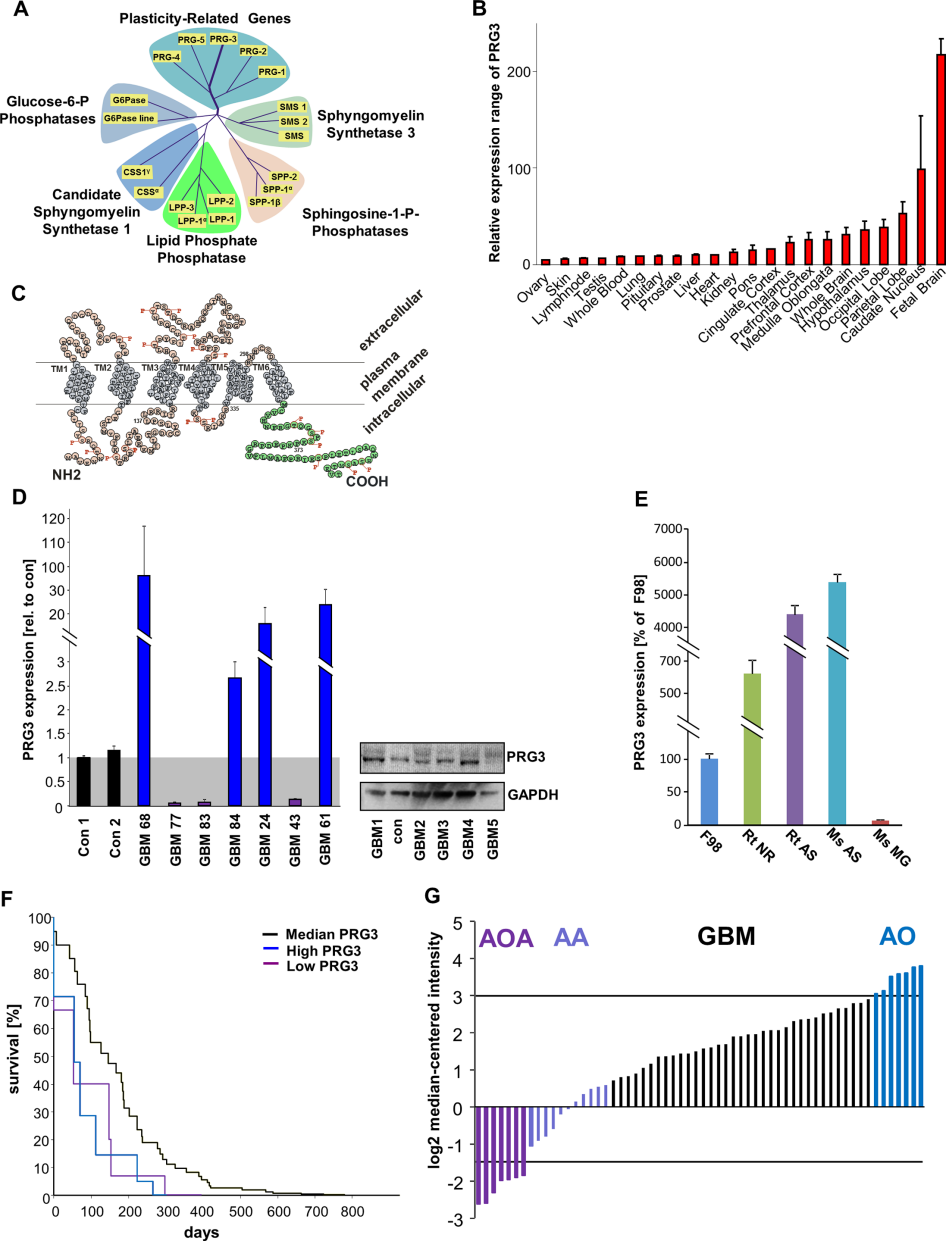
PRG3 expression is perturbed in human malignant gliomas and is associated with poor outcome (**A**) Phylogenetic analysis of the lipid phosphate phosphatases superfamily consisting of the Plasticity related gene (PRG/LPRP) family with the particular member PRG3. PRG3 is mostly related to PRG5 within the PRG family. (**B**) BIO-GPS expression analysis of PRG3 in healthy human tissue. Expression of PRG3 is high in different brain areas and highest in the developing brain. (**C**) Proposed orientation of PRG3 in the plasma membrane. PRG3 has six transmembrane domains. The 52 amino-acid long C-terminus is located intracellular (marked in green). Single circles represent amino acids (one letter code). (**D**) Human malignant glioma specimens (GBM) show a broad variability of PRG3 expression levels at the mRNA and protein level. *Left*, quantitative RT-PCR analysis for PRG3 from patients who underwent neurosurgery. Values are given relative to control brain tissues free from any malignant neoplastic affection, value of con1 was normalized as 1 and underlined in grey. *Right*, PRG3 protein analysis from various human GBM samples. (**E**) PRG3 mRNA expression in rodent and murine cells, Expression is shown in rodent glioma cells (F98), rat primary neurons (Rt NS), rat and mouse primary astrocytes (Rt AS, Ms AS) and murine primary microglial cells (Ms MG). (**F**) Human survival according to perturbed PRG3 levels in patients with glioblastoma (Oncomine data bank analysis, Freije brain library). Note that higher (> 3) and lower (< −1.5) levels of PRG3 compared to median PRG3 probe sets are associated with shorter survival. Controls are given in black (*n* = 41), high PRG3 glioblastomas are given in blue (*n* = 6) and low PRG3 glioblastomas are given in purple (*n* = 5). (**G**) Oncomine database analysis (Freije Brain library) of different human primary brain tumor entities. AA, anaplastic astrocytoma (*n* = 8); AOA, anaplastic oligoastrocytoma (*n* = 7); AO anaplastic oligodendroglioma (*n* = 11); GBM, glioblastoma (*n* = 59). Note, that within the glioblastoma group two subgroups appear with PRG3 expression at high levels as well as with low PRG3 levels compared to the average expression levels.

The PRG family has been identified during the course of differential gene expression analysis for neuroregeneration-associated molecules [12]. Currently, the family consists of five members closely related to the lipid phosphate phosphatase superfamily [21] (Figure 1A). PRG3 transcript is highly conserved among human, rodent and murine genomes (Figure S1). Analysis of PRG3 revealed its high expression during neuronal development [13, 16]. Further investigations display brain tissue as the source with the highest PRG3 mRNA levels, and low expression levels in peripheral tissues such as lung, heart and liver (Figure 1B). Although structurally related to lipid phosphate phosphatases, PRG3 does not bear phosphatase-active domains at its extracellular domains (Figure 1C). We next analyzed the levels of PRG3 in human glioblastoma samples. In comparison to non-cancerous transformed human tissue human GBMs show altered PRG3 levels ranging from up to 30 fold increased expression to reduction below 10% of human brain tissue controls (Figure 1D). Further analysis revealed opposing PRG3 levels in human GBM specimens showing high levels as well as reduced PRG3 also at the protein level (Figure 1D). We further analyzed PRG3 expression in rodent and murine primary astrocytes and neurons in comparison to glioma cells. Interestingly, murine and rodent astrocytes displayed higher PRG3 mRNA expression levels compared to neurons or F98 glioma cells (Figure 1E). Oncomine databases on survival of patients suffering from glioblastomas (GBM) showed that disturbed PRG3 levels (> 3 and < −1.5 from normalized log2 median level) are associated with shorter survival outcome compared to human GBM patients with median PRG3 expression (Figure 1F). Moreover, expression analysis revealed opposing PRG3 levels in human malignant gliomas (glioblastoma multiforme, GBM) with specimens showing high levels as well as reduced PRG3 levels (Figure 1G).

### Imbalanced PRG3 promotes cell growth and reduces apoptosis

**Figure 2:**
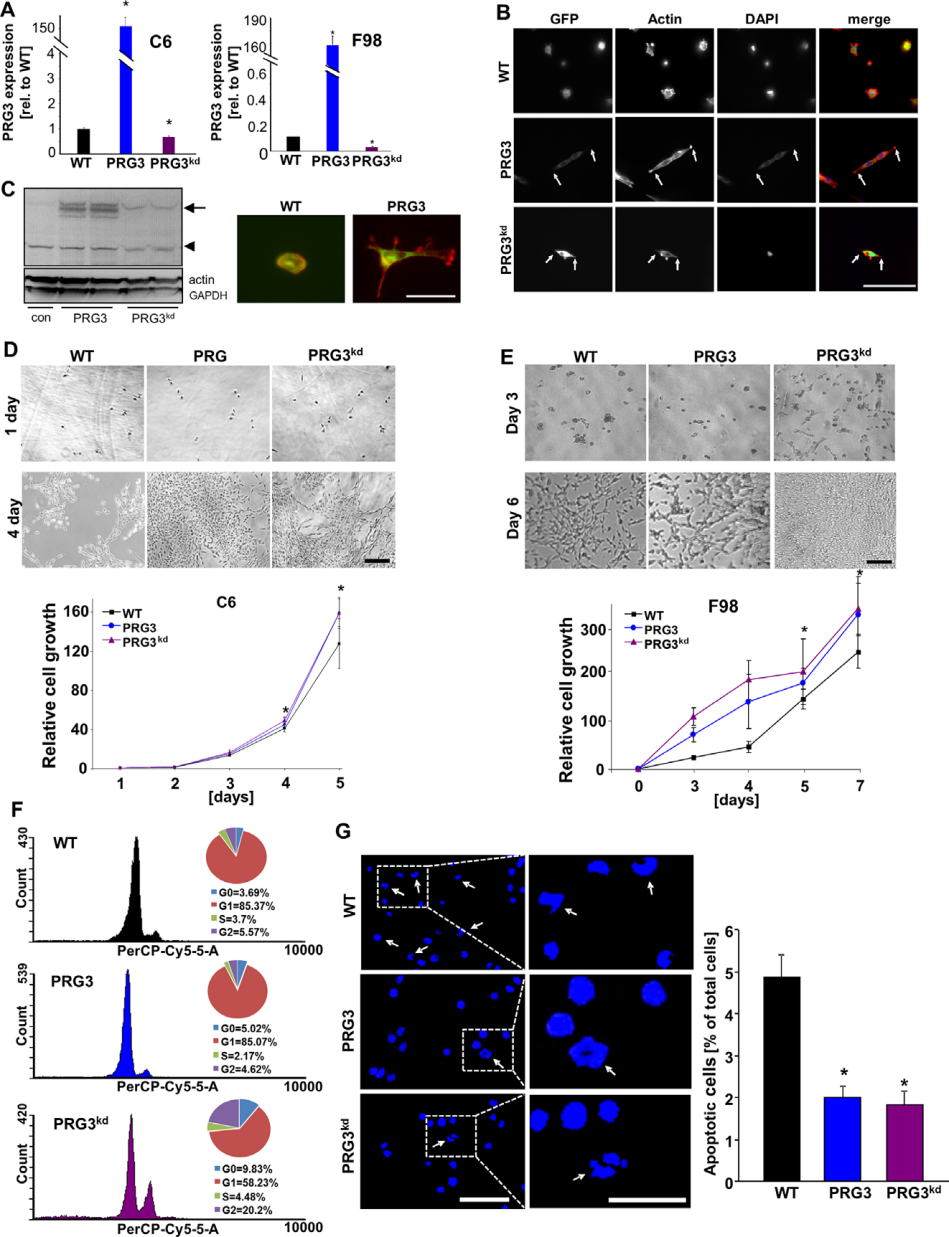
Distinct threshold of PRG3 promotes cell growth and reduces apoptosis (**A**) Quantitative RT-PCR analysis for determining PRG3 transcription in C6 and F98 glioma cells. (**B**) Morphological changes of wild type glioma cells (F98) expressing GFP (WT), PRG3 overexpression (PRG3) and RNAi mediated PRG3 knock down (PRG3^kd^). Scale bar represents 100 μm. Note, that PRG3 overexpressing and PRG3^kd^ gliomas reveal similar phenotypes and give rise to elongated and fusiform cell shapes in contrast to wild type gliomas cells (WT) with more round cell shape. (**C**) Left, Western blot showed PRG3 protein level of wild type (WT) glioma cells, PRG3 overexpression (PRG3) and RNAi mediated PRG3 knock down (PRG3^kd^). Right, Expression of PRG3 induces cell-type specific effects. Human glioma cells expressing PRG3 show an elongated morphology. Scale bar indicates 50 μm. (**D**) Deregulated PRG3 expression increases glioma cell proliferation. Top, Representative images from time-lapse experiments are given showing wild type glioma cells (WT), PRG3 overexpressing (PRG3) and PRG3 knockdown gliomas (PRG3^kd^) (rat C6 glioma cells) at different time-points (day 1–5). Scale bar represents 400 μm. Bottom, Quantitative analysis of cell growth over time. Cell growth rates of GFP+ wild type C6 rat glioma cells (WT), PRG3 overexpression (PRG3) and PRG3 knockdown glioma cells (PRG3^kd^). Statistical analysis was performed with students *t*-test (*n* = 8), values are mean ± SD, *P < 0.001. (**E**) Top, Representative images of glioma cells (F98) with low (PRG3^kd^), intermediate (WT) and high (PRG3) PRG3 expression levels assessed three days and 6 days in culture. Control-transfected wild-type gliomas proliferate significantly slower than PRG3 overexpressing and knockdown gliomas. Scale bar represent 200 μm Bottom, cell growth rates of rat F98 gliomas showing significant differences between glioma cells expressing different levels of PRG3. Overexpression and knockdown gliomas grow significantly faster than wild-type glioma cells. Statistical analysis was performed with students *t*-test (*n* = 8 per group), given values are mean ± SD, *P < 0.001. (**F**) Cell cycle analysis in rat glioma cells (F98) with low (PRG3^kd^), intermediate (WT) and high (PRG3) PRG3 expression. Representative graphs are shown on the left. Circle diagrams on the right show percentage of cells in different cell cycle phases indicated on the left (*n* = 3). (**G**) Apoptosis in glioma cells with low (PRG3^kd^), intermediate (WT) and high (PRG3) PRG3 expression levels. Left, the extent of apoptosis was evaluated by morphological analysis. Representative images of low (PRG3^kd^), intermediate (WT) and high (PRG3) PRG3 expressing gliomas stained for nuclei with Hoechst 33342. Arrows point to fragmented nuclei, which were assessed as an indicator for apoptosis. Right, quantification of apoptosis bodies. Scale bar, 50 μm. Differences were considered statistically significant with values given as mean ± SEM (*n* = 10 per group; unpaired *t*-test, *P < 0.001).

We next tested whether perturbed PRG3 thresholds as found in human GBMs have an impact on glioma growth and apoptosis. First, well established murine, rodent and human glioma cells were utilized and deregulated PRG3 expression was induced (Figure 2A, Figure S2). We determined the expression of C6 and F98 glioma cells with disturbed PRG3 levels by quantitative RT-PCR analysis. Transcriptional analysis revealed a more than 50% reduction in PRG3 knockdown gliomas and a 150 fold PRG3 mRNA increase in PRG3 overexpressing gliomas (Figure 2A). Thereafter, we analyzed the cellular effects of various PRG3 levels in glioma cells. PRG3 overexpression as well as RNAi mediated PRG3 knock down altered F98 glioma cell morphology with elongated and fusiform cell shapes in contrast to control glioma cells giving a more polygonal morphology (Figure 2B). We could monitor these morphological shapes also in human glioma cells (Figure 2C). Thus, distinctly altered PRG3 levels are associated with malignant gliomas in humans, and challenge glioma cell shape. Further, cell growth was monitored in time-lapse mode and revealed that PRG3 overexpressing gliomas grow faster than mock vector transfected gliomas (named as wild type, WT) (Figure 2D, 2E, Figure S2). However, also PRG3 knockdown cells conferred an increased growth potential by contrast to parental wild type cells (Figure 2D, 2E). Comparisons of PRG3 overexpressing, knockdown, and wild-type gliomas revealed that down-regulated PRG3 expressing gliomas grow significantly faster than wild-type and PRG3 overexpressing glioma cells (Figure 2E). We then investigated whether an imbalanced PRG3 expression in gliomas affect cell cycle and cell death. Cell cycle profiling discerned that high PRG3 expression did not significantly alter cell cycle progression compared with wild type glioma cells. However, ablated PRG3 expression altered the cell cycle, and we found that the proportion of cells in G_0_ and G_2_ phase significantly increased compared to wild type tumors. (Figure 2F). Interestingly, glioma cells with up and down regulated PRG3 both showed reduced apoptosis compared to wild-type gliomas (Figure 2G).

### Perturbed PRG3 expression promotes glioma migration and oncogenesis

**Figure 3:**
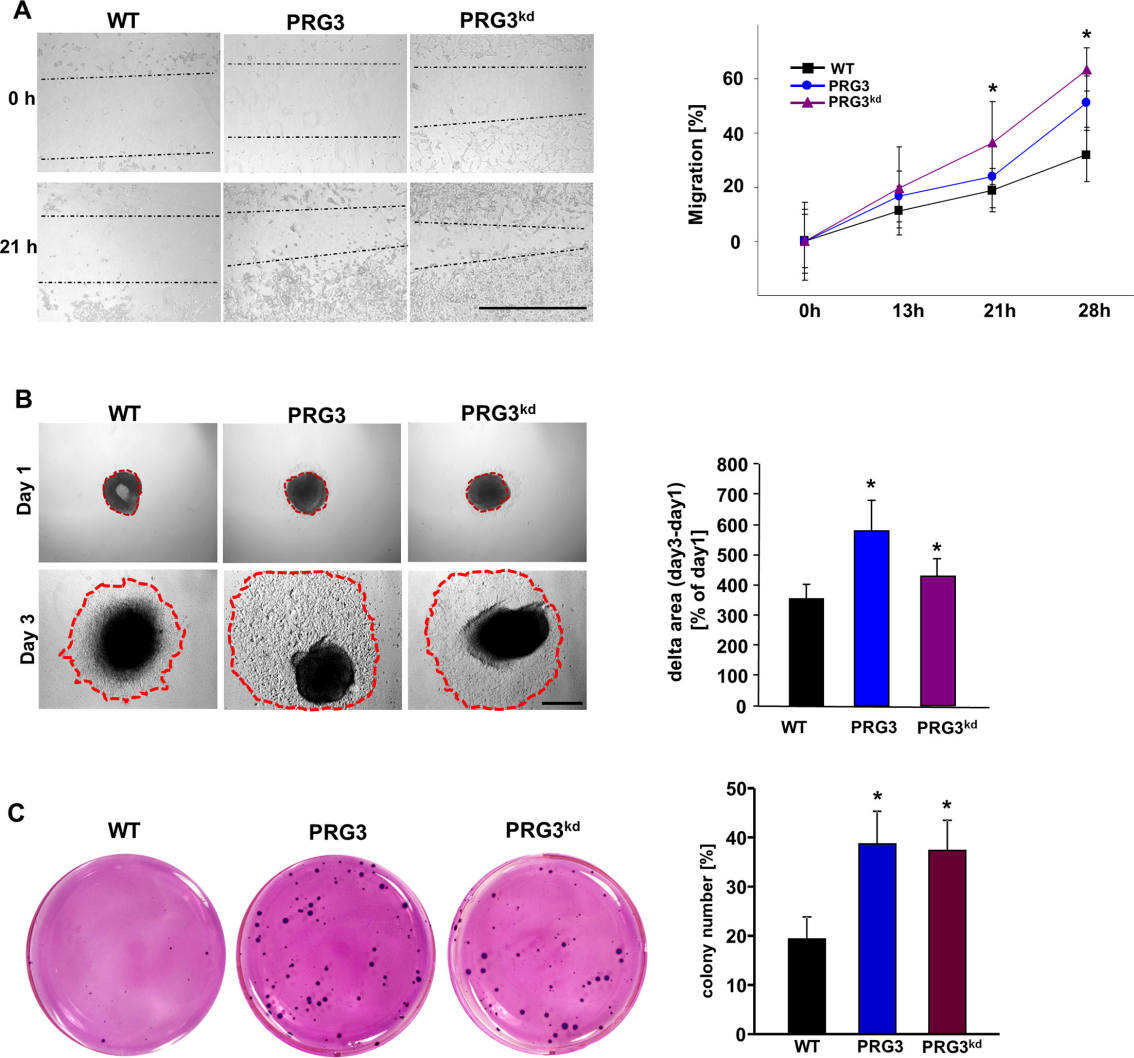
Deregulated PRG3 expression promotes gliomas migration and oncogenic transformation (**A**) Time-lapse measurements of glioma cell migration. Left, representative images of glioma cells from wild type (WT), PRG3 overexpression (PRG3) and RNAi mediated PRG3 knock down (PRG3^kd^) after 0, 13, 21, and 28 hours. Dotted lines indicate migrating cell fronts. Scale bar indicates 1000 μm. Right, quantitation of the time-lapse migration assay. Note that glioma cells with low (PRG3^kd^) and high (PRG3) PRG3 expression levels migrate faster and have greater ability to close the scratch than wild type gliomas (WT). Three independent experiments were carried out and differences were considered statistically significant with values given as mean ± SD (*n* = 6; *t*-test, *P < 0.001). (**B**) Glioma cell migration monitored via sphenoid assay. Left, representative images of F98 glioma cell clusters from wild type (WT), PRG3 overexpression (PRG3) and RNAi mediated PRG3 knock down (PRG3^kd^) after day 1 and day 3. Dotted red lines indicate glioma cell migration fronts. Scale bar indicates 1 mm. *Right*, quantitation of the sphenoid assay. Note that glioma cells with low (PRG3^kd^) and high PRG3 expression levels migrate faster and stronger than wild type gliomas (WT). Three independent experiments were carried out and differences were considered statistically significant with values given as mean ± SD (*n* = 6; *t*-test, *P < 0.001). (**C**) Oncogenic amplification of glioma cells after PRG3 deregulation. Left, representative images of colonies formed in soft agar from wild-type glioma cells (WT), PRG3 overexpressing gliomas (PRG3) and PRG3 knockdown glioma cells (PRG3^kd^). Right, quantitative analysis of colony formation of glioma cell clusters bigger than 100 μm in soft agar. Quantification is given for *n* = 3. Data are given as means ± SD, *P < 0.001 (unpaired Student's *t*-test).

We further investigated whether distinct PRG3 levels impact also other malignancy characteristics such as invasive growth and anchorage-independent proliferation. Initially, we monitored glioma migration and found that PRG3 overexpressing as well as PRG3-silenced gliomas showed accelerated migration (Figure 3A). In the sphenoid assay it became apparent that both PRG3 and PRG3^kd^ tumors invade stronger into the extracellular space in comparison to wild type tumors (Figure 3B). Moreover, imbalanced PRG3 expression caused increased colony formation and growth in comparison to wild-type glioma cells (Figure 3C). In addition, deranged PRG3 in gliomas formed also bigger colonies compared to controls, thereby increasing the capability for anchor-independent growth (Figure 3C). These *in vitro* assays indicate that perturbed PRG3 expression in gliomas foster oncogenesis and increase their malignancy potential.

### Distinct thresholds of PRG3 drive glioma invasion and tumor expansion *ex vivo* and *in vivo*

**Figure 4:**
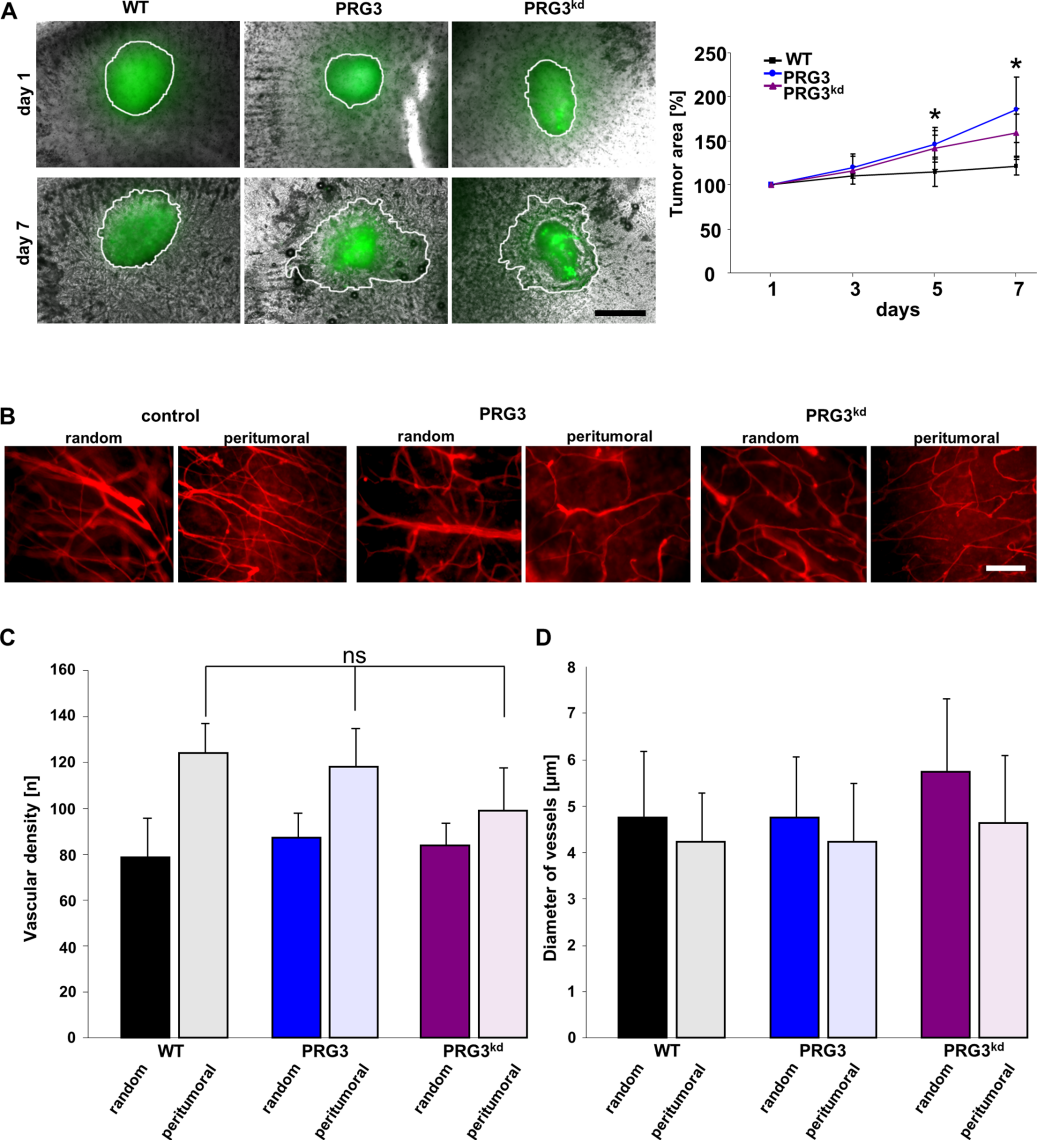
Imbalanced PRG3 levels drive glioma invasion but not tumor angiogenesis (**A**) Left, Deregulated PRG3 levels foster tumor expansion into brain parenchyma. GFP+ glioma wild type cells (WT), PRG3 expressing gliomas (PRG3) and PRG3 knockdown glioma cells (PRG3^kd^) were implanted in brain slices (grey), and tumor expansion was evaluated after 1, 3, 5, and 7 days. Representative images of tumor implanted brain slices are shown on the left. Tumor borders are outlined (white line). Scale bar indicates 500 μm. Right, Quantitative analysis of tumor growth and expansion. Black square represents wild-type glioma cells (WT); blue dot, PRG3 expressing gliomas (PRG3); purple triangle, PRG3-knockdown gliomas (PRG3^kd^). Values are given as means ± SD with *n* = 5 per group. Statistical significance was calculated with Student's *t*-test (mean ± SD, *P < 0.05). (**B**) Relation of perturbed PRG3 expression and tumor angiogenesis. Representative images of vessels in random control regions and peritumoral zones of control gliomas (WT), PRG3 expressing gliomas (PRG3), and PRG3-knockdown gliomas (PRG3^kd^). Scale bar indicates 100 μm. (**C**) Quantitative analysis of tumor angiogenesis in wild-type gliomas (WT), PRG3 expressing gliomas (PRG3), and PRG3-knockdown gliomas (PRG3^kd^). Data are given as means ± SD, *n* = 4 per group including 4 regions for peritumoral and control regions per slice. Statistical significance was calculated with Student's unpaired *t*-test (values are given as mean ± SD, n.s., not significant).

We continued to test whether PRG3-driven malignancy promotes glioma growth also in the context of a complex brain microenvironment including all cellular constituents. For this purpose we implanted syngeneic gliomas into brain tissue and monitored their growth *ex vivo*. Gliomas with imbalanced PRG3 expression showed stronger infiltrative tumor expansion into brain parenchyma with a polarized and irregular tumor front compared to wild-type gliomas (Figure 4A). In addition, the overall tumor growth and infiltrative brain area was higher than in wild-type gliomas (Figure 4A). Since one malignancy criterion in human gliomas is the extent of tumor vasculature we further analyzed angiogenesis in gliomas. Wild-type gliomas induced augmented vessel growth compared to unaffected brain areas (Figure 4B). Interestingly, PRG3 overexpressing and PRG3 silenced gliomas showed comparable vascular density to wild-type gliomas (Figure 4B, 4C). Also, diameter of vessels were comparable among these tumor groups (Figure 4D). Although these data are consistent with the hypothesis that imbalanced PRG3 expression in gliomas amplifies their malignancy, it is conceivable that this tumor growth may not sustainable *in vivo* due to its altered angiogenesis.

**Figure 5:**
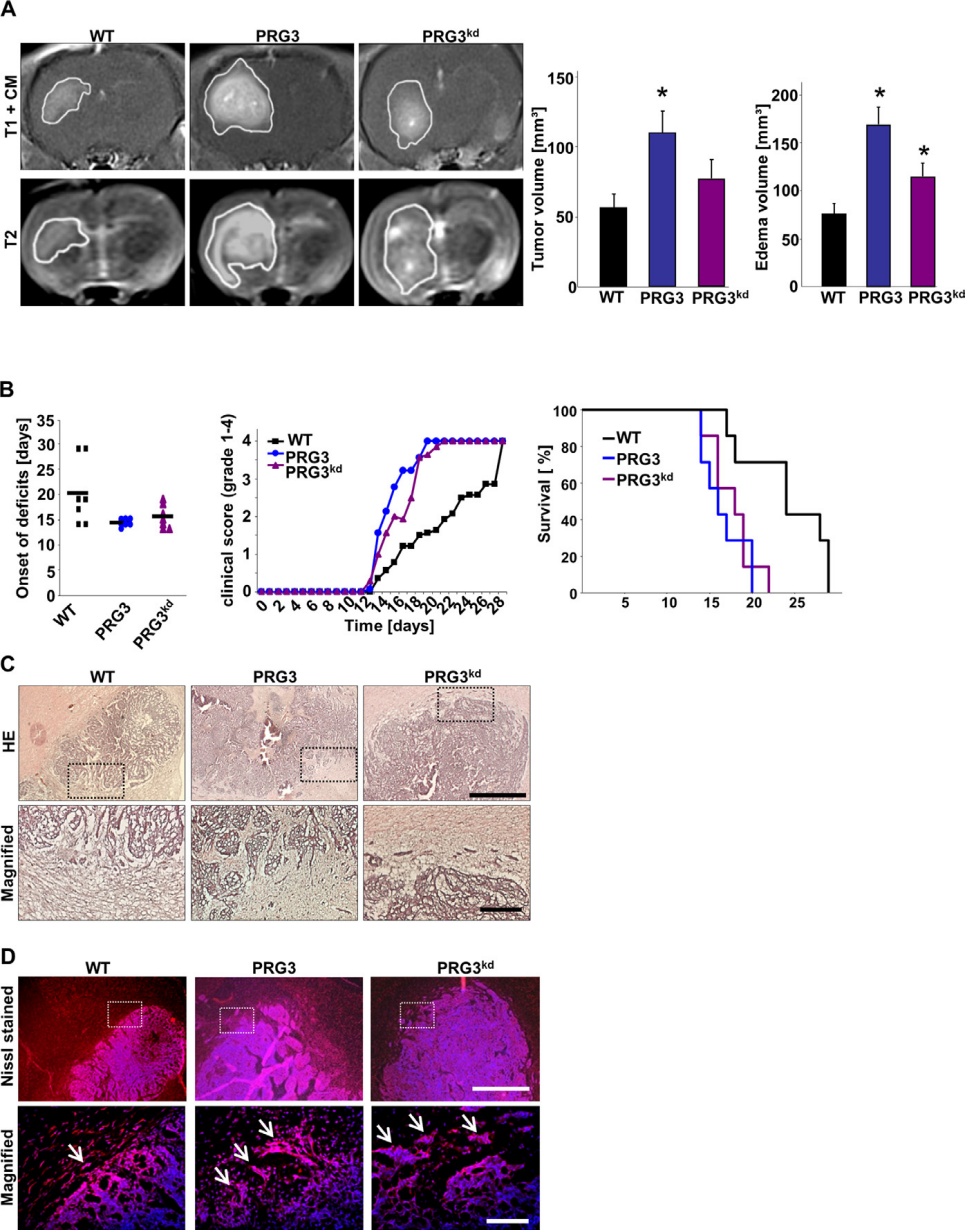
Imbalanced PRG3 levels drive glioma expansion and brain edema (**A**) Deregulated expression of PRG3 in gliomas accelerates tumor growth and the onset of neurological deficits *in vivo*. Representative MR images of wild-type (WT), PRG3 overexpressing (PRG3) and PRG3-knockdown (PRG3^kd^) gliomas 10 days after tumor implantation in male Fisher rats weighting approximately 180–200 g. The tumor bulk (marked with continuous white line) was visualized after application of intraperitoneal contrast agent and subsequent T1-weighted imaging (upper row, T1+CM). *Bottom*, corresponding T2-weighted images of brains from wild-type (WT), PRG3 overexpressing (PRG3) and PRG3-knockdown (PRG3^kd^) gliomas. The marked area indicates total tumor volume (including peritumoral and edema zone). *Right*, Quantification of tumor volume out of T1 and T2-weighted MR images from wild-type (WT), PRG3 overexpressing (PRG3) and PRG3-knockdown (PRG3^kd^) gliomas. Tumor volume and peritumoral edema zone was significantly smaller in control wild-type (WT) gliomas compared to PRG3 overexpressing (PRG3) and PRG3-knockdown (PRG3^kd^) gliomas. Statistical significance was calculated with Student's *t*-test (mean ± SEM, *P < 0.05). (**B**) Animals were clinically assessed on a daily basis and evaluated according to their neurological status (grade 0: normal; grade 1: tail weakness or tail paralysis; grade 2: hind leg paraparesis or hemiparesis; grade 3: hind leg paralysis or hemiparalysis; grade 4: complete paralysis (tetraplegia), moribund stage or death) and onset of neurological symptoms were measured. Note, that animals with PRG3 overexpressing (PRG3) and PRG3-knockdown (PRG3^kd^) gliomas develop earlier neurological deficits compared to animals with wild-type (WT) gliomas. *Middle*, Clinical progression of neurological deficits in rats bearing wild type gliomas (black squares) or oncogenic transformed glioma cells with PRG3 overexpression (blue dots) or PRG3 silencing (purple triangles). *Right*, Kaplan Meier survival curves of rats implanted with wild-type (WT), PRG3 overexpressing (PRG3) and PRG3-knockdown (PRG3^kd^) gliomas. Statistical significance was calculated with Student's *t*-test (mean ± SD, *P < 0.05, *n* = 7 per group). (**C**) *Top*, Representative histological sections stained for HE from wild-type gliomas (WT), PRG3 gliomas (PRG3), and PRG3 Knock down gliomas (PRG3^kd^). White dashed boxes indicate the tumor border. Scale bar represents 500 μm. *Bottom*, higher magnification of the area in white dashed boxes. Scale bar represents 50 μm. (**D**) *Top*, Representative immunofluorescence images for Nissl stained (red) cryosections in the tumor area from wild-type gliomas (WT), PRG3 gliomas (PRG3), and PRG3 Knock down gliomas (PRG3^kd^). Nuclei are stained with Hoechst and given in blue. White dashed boxes indicate the tumor border. Scale bar represents 500 μm. *Bottom*, higher magnification of the area in white dashed boxes. Scale bar represents 50 μm.

To test whether imbalanced PRG3 levels are advantageous and long-lasting we analyzed glioma growth *in vivo* by orthotopic implantation of syngeneic glioma cells into rat brains. *In vivo* tumor monitoring by contrast-enhanced T1-weighted 3 Tesla magnetic resonance imaging (MRi) 10 days after implantation further revealed increased tumor volume in PRG3 imbalanced gliomas (Figure 5A). T2-weighted images further demonstrate augmented perifocal brain edema in PRG3 overexpressing and silenced gliomas compared to wild-type gliomas (Figure 5A). Statistical analysis revealed that both PRG3 overexpressing and PRG3 silenced gliomas showed an accelerated onset and progression of neurological deficits compared to control tumors (Figure 5B). To further test whether the boosted tumor growth rates have any sustainable clinical effects we analyzed survival prolongation. Animals bearing gliomas with imbalanced PRG3 expression showed reduced overall survival compared to wild-type glioma bearing animals (Figure 5B). We next performed histological analysis on brain sections from WT, PRG3 and PRG3^kd^ glioma implanted animals. Thereby, we found that both PRG3 and PRG3^kd^ tumors have larger volume in comparison to wild type tumors (Figure 5C). Moreover, the tumor margins in wild type tumors were less diffuse compared to PRG3 tumors (Figure 5C, 5D). In PRG3 and PRG3^kd^ glioma implanted brains, the tumor border displayed a diffuse margin with an invasive pattern (Figure 5D). Moreover, the Nissl staining specific for neuronal cells revealed peritumoral damage in all tumor entities (Figure 5D). These findings corroborate the *in vitro* cell growth data showing that imbalanced PRG3 levels accelerate tumor growth *in vivo*.

### PRG3-induced effects are mediated via PRG3-RasGEF1 interaction and Ras activation

**Figure 6:**
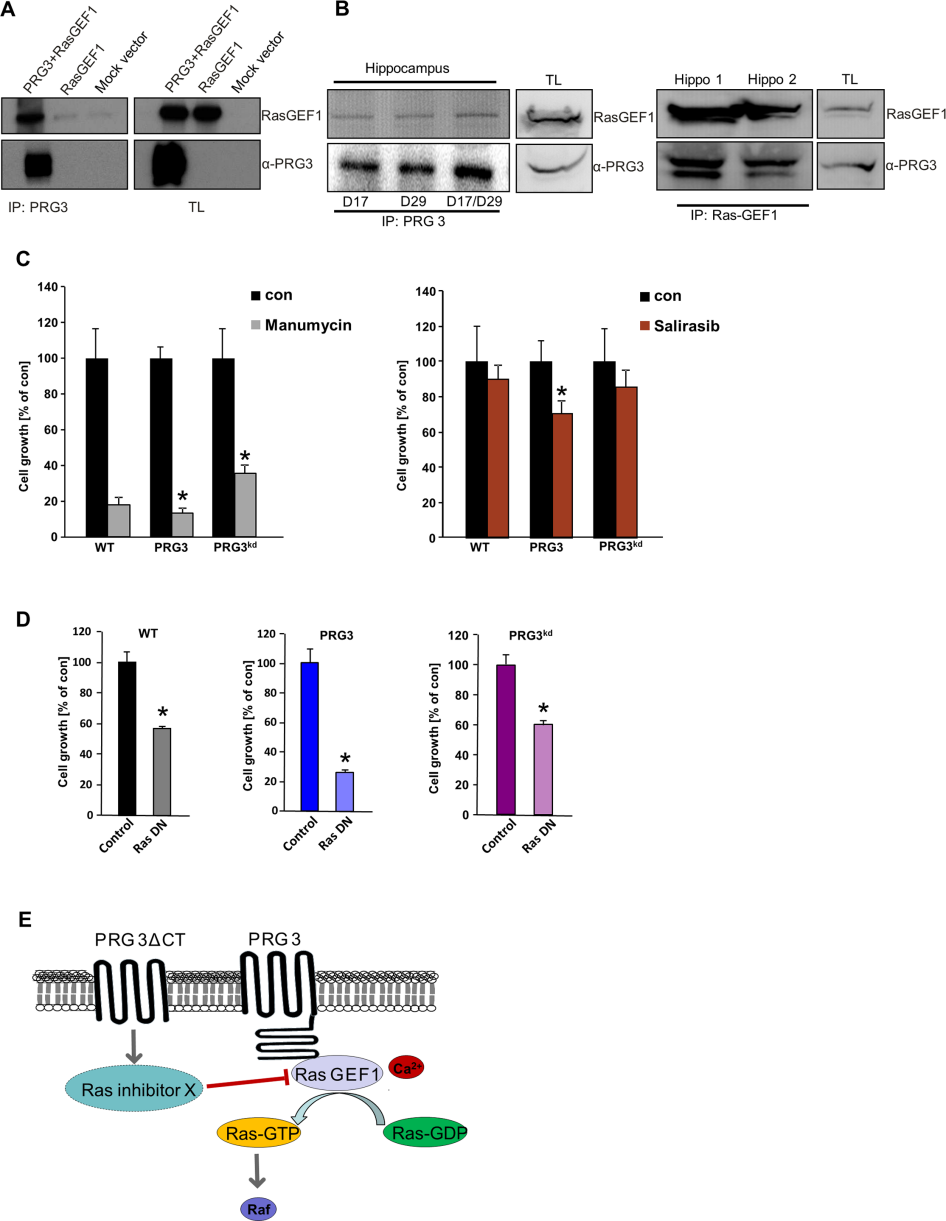
Oncogenic effects of imbalanced PRG3 are mediated via PRG3-RasGEF1 interaction and Ras activation (**A**) Left, PRG3 interacts with RasGEF1. Cells were transfected with either flag-tagged PRG3 plus RasGEF1, solely RasGEF1 or mock vector and immunoprecipitated for PRG3. Right, immunoblots of total lysates (TL) are shown for RasGEF1 and PRG3 expression controls. (**B**) PRG3 interacts with RasGEF1 *in vivo*. Left panel shows endogenous PRG3/RasGEF1 complex detected by immunoprecipitation with various PRG3 antibodies in different brain tissue samples (hippocampus). Right panel shows endogenous PRG3/RasGEF1 complex detected by immunoprecipitation with RasGEF1. (**C**) PRG3 and PRG3^kd^ glioma cells response differently to Ras inhibitors. Quantitative analysis of cell growth revealed that Ras inhibitor manumycin (5 μM) and salirasib (20 μM) inhibit PRG3 glioma cell growth by 86% and 30% respectively, while reduced PRG3^kd^ gliomas proliferation by 64% and 14% respectively. Non-treated control of each cell types (con) were set as 100% and given in black. Statistical significance was calculated with Student's *t*-test (mean ± SD, *n* = 8 per group; *P < 0.05). (**D**) PRG3 glioma cells response differently to dominant negative Ras (RasN17) compared to wild type (WT) and PRG^kd^ gliomas. Quantitative analysis of cell growth revealed that dominant negative Ras inhibits PRG3 glioma cell growth by 28% whereas PRG3^kd^ gliomas and WT gliomas are less sensitive. Non-treated control of each cell types (con) were set as 100% and given in black. Statistical significance was calculated with Student's *t*-test (mean ± SD, *n* = 8 per group; *P < 0.05). (**E**) PRG3 signaling domains and downstream consequences. Summary of PRG3 signaling domains and their effects on Ras activation. Full length PRG3 is a multidomain protein possessing an intracellular C-terminal tail interacting with the guanine nucleotide exchange factor RasGEF1 (blue circle). The integral membrane domain of PRG3 is proposed to decrease Ras activation.

To determine how tumor growth is regulated in PRG3 disequilibrated gliomas we analyzed possible intracellular interaction partners using the C-terminal tail as bait. Verification hits out of this yeast two-hybrid screen revealed that the Ras GTPase exchange factor RasGEF1 (RasGRF1/GRF1) interacts with PRG3 (Figure 6A). Further, the endogenous PRG3-RasGEF1 complex could further be confirmed *in vivo* in protein lysates from brain tissue. In hippocampus tissue we found that endogenous PRG3 co-immunoprecipitates with RasGEF1 (Figure 6B). Since RasGEF1 acts as an oncogene in various malignant tumors by regulating the GTPase Ras [22, 23], we further tested the downstream signals in gliomas. Interestingly, PRG3 overexpressing and PRG3 silenced gliomas conversely increased oncogenic Ras activation compared to wild-type gliomas (Figure S3A). We consequently investigated the domains essential for PRG3 signaling. For this we tested in particular the C-terminal domain (PRG3^CT^) due to its ability to interact with RasGEF1. In addition, we compared the Ras activity of PRG3 constructs lacking the C-terminal domain. Deletion of the C-terminal domain in PRG3 (PRG3^ΔCT^) led to decreased Ras activation compared to control gliomas (Figure S3A). These findings indicate that imbalanced PRG3 levels in gliomas cause Ras-dependent amplification [24]. Expression of PRG3^CT^ in gliomas amplified proliferation and transformed cellular morphology comparable to PRG3 full length expressing gliomas (Figure S3B, S3C). We also investigated the anchor-independent growth in the context of the C-terminal domain of PRG3. PRG3^CT^ expression conferred glioma cells increased formation of colonies compared to wild-type gliomas (Figure S4). Moreover, gliomas expressing PRG3^CT^ revealed increased Ras activation in comparison to parental cells (Figure S3C). These data indicate that PRG3-induced Ras is mediated through its intracellular C-terminal domain.

We further tested the functional dependence of PRG3 on Ras. For this we treated WT, PGR3 and PRG3^kd^ glioma cells with specific Ras inhibitors (manumycin and salirasib) and monitored cell proliferation. These experiments revealed that gliomas with elevated PRG3 levels were significantly more sensitive to Ras inhibition compared to parental tumor cells and PRG3^kd^ gliomas (Figure 6C). Moreover, PRG3^kd^ gliomas showed a certain Ras-independency compared to parental gliomas at least in the case of manumycin (Figure 6C). Thus, PRG3 expressing tumors mediate Ras-dependent oncogenic effects whereas reduced PRG3 levels act independent of RAS activation. We further tested whether the proliferation of glioma can be affected when Ras is down-regulated. For this approach we used a dominant negative Ras construct (RasN17) and compared the cell growth rate to controls (Figure 6D). Notably, PRG3 expressing gliomas were highly sensitive towards Ras inhibition in comparison to PRG3^kd^ gliomas (Figure 6D). These data support the conclusion that PRG3 mediate Ras-dependent oncogenic effects whereas PRG3^kd^ gliomas act independently of Ras.

## DISCUSSION

In this study we investigated the impact of the PRG3 on glioma morphology and function. Our results demonstrate that perturbed levels of the primarily neuronal integral membrane protein PRG3 can promote oncogenesis in glioma cells. Thus, its fine-tuned regulation of PRG3 under physiological conditions during development indicates its importance for tightly controlled homeostasis and function [13, 17].

Further, our data indicate that homeostatic imbalances in PRG3 levels constitute an oncogenic driver which confers selective growth advantage. Initial experiments in neurons, however, reveal no growth promoting effects in primary neurons after ectopic PRG3 expression or PRG3 gene silencing. Since even fully differentiated neurons and astrocytes can give rise to full-blown malignant gliomas [10], genetic lesions affecting PRG3 levels directly or via transcriptional regulation may contribute to oncogenic cooperation in the process of aggravated malignancy. It has been shown that dependent on the culture protocol solely up to 4 factors are required to induce pluripotency even in fully differentiated adult neurons [25, 26]. However, in contrast to embryonic stem cells induced pluripotent stem cells share also common pathways and gene expression pattern related to tumorigenicity [27]. These alterations affect mainly the physiological expression threshold rather than gain or loss-of-function shifts [28].

Thus, imbalanced expression thresholds of tumor suppressors and oncogenes such as myc, ras, and p53 cooperate in oncogenesis and tumorigenesis [29–32]. It is conceivable that oncogenic cooperation in single tumor cells could be one mechanism by which imbalanced PRG3 executes aggravated malignancy in gliomas. Our data indicate that PRG3 appears as a new factor involved in differentiation and de-differentiation processes. The fine-tuning of PRG3 levels at a balanced threshold is important in maintaining fundamental cell functions such as differentiation, proliferation and migration. The importance of PRG3 regulation has been shown in the nervous system, where disrupted PRG3 levels are associated with epileptic seizures and neurite growth [13, 20]. What remains is why a neuronal-associated gene is expressed at all in transformed glial cell populations. Interestingly, evidences exist that support the heterogeneity of gliomagenesis arising from fully differentiated glial cells as well as neurons [10]. Hence, gliomas with a neuronal expression signature exhibits strong malignancy which indicates that neural genes such as PRG3 operate in tumor progression and amplified oncogenic signaling. Further studies will relate to the molecular signature of PRG3-derived tumors, i.e. whether various perturbations of PRG3 levels cause different subtypes of gliomas, i.e. such as the development of mesenchymal or neural glioblastoma subtypes [5, 33]. Recent genome-wide association studies already revealed the significance of such expression signature. These analyses demonstrated that within the same histopathological entity of glioblastomas (°IV according to the WHO classification) different glioma subtypes are captured with probably differential responsiveness to chemotherapeutic agents [6, 34]. Further, particular perturbed gene expression levels have been shown to influence the clinical outcome in tumor diseases. Differential expression levels such as of MGMT, TP53, EGFR, and IDH mutations showed already predictive values for neuro-oncological therapies and thus have currently become part of routine molecular diagnostics [35].

The clinical relevance to our findings is further supported by clinical data base studies on malignant gliomas, where PRG3 is expressed in opposing amounts in the way that either PRG3 is elevated in human samples or repressed compared to non-transformed human specimens. Experimental data support the human expression traits of PRG3 in malignant gliomas. This oncogenic behavior comes close to the deregulation of transcription factors: a change in expression, whether up- or down-regulation, leads to oncogenic acceleration. This novelty is astonishing, as PRG3 is a member of a PRG family up to now known to operate in neuronal differentiation and transmission. Oncogenic cooperation is a common mechanism facilitated by tumor cells and has been shown for Myc, Ras, and p53. Disturbed levels of these factors or perturbed genes dosage make cells prone to undergo transformation [30, 31, 36, 37].

What is the operational mode of PRG3 in gliomas? Investigations of the underlying signaling pathways revealed that PRG3 interacts with RasGEF1 and activates Ras possibly through the lipid second messenger PIP_2_. This is further evidenced by responses to Ras inhibition in PRG3 expressing tumor cells. Moreover, diminished PRG3 levels led also to Ras activation although these tumor cells are not prone for Ras inhibition. This indicates that similar downstream targets can be affected through imbalanced PRG3 levels with different biological consequences. We could further show that PRG3 executes the binding to RasGEF1 predominantly via its C-terminal domain (CT) and in the consequence causes Ras activation. Moreover, deletion of the CT of PRG3 abolished the Ras activating effects (Figure 6E).

A recent study further indicated that PRG3 preferentially interacts with PRG1, although binding of PRG3 was shown with all PRG family members [38].

The significance of deranged PRG3 levels is reflected by the response rates to therapeutic small molecule inhibitors. Thus, gliomas with high PRG3 levels are prone to Ras blockers and their tumor growth can be treated with Ras-inhibiting drugs such as salirasib. Future studies will unravel whether other kinases are affected by PRG3, and if so in which hierarchy the downstream targets affect cell growth. Altogether, we provide evidence that PRG3 acts dosage and context-dependent in the nervous system, and interference with its balanced level can cooperate in oncogenic signaling.

## MATERIALS AND METHODS

### Cell lines, transfection and immunoblotting

Rodent glioma cell lines F98, C6, and the human glioma cell line U87 were obtained from ATCC/LGC-2397 (Germany) and were cultured under standard condition containing DMEM medium (Biochrom, Berlin, Germany) supplemented with 10% fetale bovine serum (Biochrom, Berlin, Germany), 1% Penicillin/Streptomycin (Biochrom, Berlin, Germany) and 1% Glutamax (Gibco/Invitrogen, California, USA). Cells were passaged at approx. 80% confluence by adding trypsin after 1 PBS wash step and incubated for 5 min, then centrifuged at 900 rpm/5 min.) Cell lines were transfected according to Broggini et al. [14]. Briefly, cells were plated at 20 000/cm² in 6-well plates and held under standard conditions. 24 hours (h) after seeding transfection was performed using Roti-Fect (Roth, Karlsruhe, Germany) according to the manufacturer's protocol. Transfected cells were selected with geneticin sulfate 418 (Sigma, St. Louis, USA) and fluorescence-activated cell sorting was performed. Stable clones were further maintained under selection and expression levels were validated by qRT-PCR. Western blot analysis was performed as described previously [39]. Cells were maintained under standard conditions. For protein extraction samples were lysed with NP 40 buffer containing a protease inhibitor cocktail (Roche, Basel, Switzerland) and homogenized by ultrasound (Bandelin Sonoplus, at 67%). After 20 min on ice, samples were centrifuged at 8000 rpm for 8 min. Supernatants were measured with Nano-drop (Thermo-scientific, Massachusetts, USA). Samples were mixed with loading buffer (4×) and Reducing agent (10×) (Invitrogen, California, USA) and boiled at 96°C for 8 min. Equal amounts of protein sample were loaded on 4–12% SDS-NuPage Gel (Invitrogen, CA, USA) and electrophoresis was performed in MOPS-buffer, transferred on PVDF membranes (Roth, Karlsruhe, Germany) and efficiency was checked with Memcode Stain kit (Thermo Massachusetts, USA) according to the user manual. Membranes were blocked in PBS containing 2% Magic block and 10% Top block (Lubio science, Lucern, Switzerland) for 1 h before further processed. Antibodies were incubated overnight at 4°C in roller tubes, followed by secondary antibodies incubated at room temperature for 1 h. Detection was performed with ECL plus kit (GE-healthcare, Solingen, Germany). Ras activation was determined by a Raf-RBD protein pull down assay (Cytoskeleton/TEBU Bio, Düsseldorf, Germany; Enzo life science, Lörach, Germany). All steps were performed on ice and the procedure was conducted in accordance to the manual. Briefly, cells were scraped in lysis buffer with a cell scraper. After lysate transfer, proteins were isolated via ultrasound and further incubated on ice for 5 min followed by a centrifugation step for 8 min (4000 rpm). Supernatants were transferred to a new tube. 25 μl of the Ras-binding domain was added to approx. 500 μl of total lysate and was incubated at 4°C overnight under end-to-end rock. Gluthathione discs were added the next day for 4 h and tumbled at 4°C. After incubation, beads were washed thrice and lysates were run on a SDS gels under western blotting conditions as outlined above. For *in vivo* analysis we used human samples gained from in-house surgery of patients undergoing GBM surgery. Cells were stored at −80°C freezers. After thawing, samples were lysed as described above.

### Yeast two-hybrid screening

For searching interaction partner of PRG3 a yeast two-hybrid screen was performed on a brain cDNA library (Stratagene, CA, USA). Briefly, PRG3 CT bait constructs were transformed into the reporter yeast strain Y187 and the resulting strains were grown on synthetic defined medium lacking either tryptophan (-trp) or leucine (-leu). Yeast growth was evaluated after seven days at 30°C.

### Real time PCR analysis

#### RNA isolation and qRT-PCR experiments

80% confluence cells in 75 cm^2^ flasks were trypsinized and collected in a 1.5 eppendorf tube then suspended in 200 μl PBS. Total RNA from each cell lines was extracted by using High Pure RNA Isolation Kit (Roche, Mannheim, Germany) as the protocol descript. For human GBM RNA isolation, 20 mg tumor tissue was cut into small pieces and crashed in 400 μl lysis/binding buffer (Roche. Cat. No. 12 033 674 001). Total RNA from each sample was extracted by using High Pure RNA Tissue Kit (Roche) as the protocol descript. RNA concentration was quantified by NanoVue^™^ Plus Spectrophotometer (GE Healthcare, UK). cDNA synthesis was performed with SuperScript^®^ III Reverse Transcriptase according to the manufacturer's manual (Invitrogen, Germany). qRT-PCR was performed with SYBR Green PCR master mix (Qiagen). The oligos used in this study are (species universal rat/mouse PRG3 forward primer: TATGCCACGATGTACATCAC; rat/mouse PRG3 reverse primer: AACAGTGGTTCCGGTACTCT. Human PRG3 forward primer: CCGCCTTATATGCCACGATG; Human PRG3 reverse primer: GGTTCCGATACTCAGAGACC. GAPDH forward primer: TGCACCACCAACTGCT TAGC; GAPDH reverse primer: GGCATGGACTGTGG TCATGA. Beta-actin forward primer: GCTCCTCCTGA GCGCAAG; Beta-actin reverse primer: CATCTGCTGGA AGGTGGACA). Real time cycling parameters: Initial activation step (95°C, 15 min), cycling step (denaturation 94°C, 15 s; annealing at 60°C, 30 s; and finally extension for 72°C, 30 s × 40 cycles), followed by a melting curve analysis to confirm specificity of the PCR. The Ct value was corrected by Ct reading of corresponding GAPDH or Beta-actin controls. Data from three determinations (means ± SEM) are expressed as relative expression level. The reaction was performed using Light Cycler 480 (Roche, Mannheim, Germany). The specificity of the PCR reaction was confirmed by agarose gel electrophoresis.

### Expression vectors and knock down vector cloning

Reverse transcription-polymerase chain reaction was used for full length cloning of PRG3 from rat, mouse and human mRNA samples as described previously [13]. For sequence alignments and homology searches of PRGs we utilized the www.ncbi.nlm.nih.gov database and A Plasmid editor software (ApE; MW Davis, Utah, USA). Transmembrane domains have been predicted using the Kyte Doolittle algorithm and all orthologous sequences of PRG3 (human, mouse and rat) are deposited at the NCBI database (Human PRG3 GenBank accession no. AY304516; Rattus norvegicus PRG3 GenBank accession no. AY299399; Mus musculus PRG3 GenBank Accession no. AY345342). For construct cloning we cloned fragments by PCR and inserted the resulting amplicons into the pEGFP (Takara, Heidelberg, Germany), and pmRFP (Kes Jalink, NKI, Amsterdam, the Netherlands) vectors. C-terminal domain of PRG3 was cloned by PCR amplification out of the full length clone and inserted the amplicon into linearized peGFP vectors. According to the criteria of Naito et al. [40] three 19-mer short interfering RNAs were chosen for RNA interference with rodent PRG3 transcripts (GenBank acc. AY299399). Cloning of the synthetic oligonucleotids into the pSuperGFP vector (pS-GFPneo; OligoEngine, Seattle, USA) was performed by digesting the empty vector with Bgl2 and EcoR1 according to the manufacturer's instruction.

### VOGiM brain slice cultures

Brain slice cultures of seven-day-old Wistar rats were prepared and maintained as previously described [41–42]. Animals were sacrificed and brains were removed and kept under ice-cold conditions. Frontal lobes and Cerebellum were dissected of the hemispheres. The remaining brain was cut into 350 μm thick horizontal slices using a vibratome (Leica VT 1000S, Bensheim, Germany). Brain slices were thereafter transferred onto culture plate insert membrane dishes (Greiner Bio One, Frickenhausen, Germany; pore size 0.4 μm) and subsequently transferred into six-well culture dishes (GreinerBioOne, Frickenhausen, Germany) containing 1.2 ml culture medium (MEM–HBSS, 2:1, 25% normal horse serum, 2% L-glutamine, 2.64 or 14.3 mg/ml glucose, 100 U/ml penicillin, 0.1 mg/ml streptomycin, 10 μg/ml insulin–transferrin–sodium selenite supplement and 0.8 μg/ml vitamin C). The slices were cultured in humidified atmosphere (35°C, 5% CO_2_). The medium was changed on the first day after preparation and from that time forward every other day over a course of 7 days. Invasion fronts were determined by quantifying the tumor infiltrating area.

### Cell growth and apoptosis analysis

Cell growth and proliferation assays were performed according to Eyüpoglu et al. [43] and Huang et al. [44]. Briefly, cells were plated at a density of 2500 cells/cm² and incubated under standard conditions. At count-point, cells were washed once with PBS, then trypsinized with 200 μl and incubated for 5 minutes and reaction was stopped with 800 μl culture medium. After thoroughly suspension samples were counted using cell counter Z2 (Beckmann-Coulter CA, USA) using 100 μl sample and 9.9 ml isotonic 2 solution.

Apoptosis assays were performed with modifications as described in Halstead et al. [45]. Therefore, cells plated at 15,000 cells/cm² on coverslips and incubated for 3 days. Samples were trypsinized and fixed with 4% PFA on ice for 10 min, then spinned down at 900 rpm for 5 min. Cells were stained with HOECHST 33342 (Invitrogen, SIGMA) [c = 16 μg/ml] for 10 min in the dark. Suspension was added to an objective glass and mounted on cover slips Pictures were taken at ×400 with Olympus ×71. Nuclei fragmentation was counted and set related to total number of cells on image. Cell proliferation was measured using the 3-(4, 5-dimethylthiazol-2-yl)-2, 5-diphenyl-tetrazolium-bromide (MTT) assay (detailed in Eyüpoglu et al. [46]). Briefly, cells were plated at 3000 cells/cm² in 96 well-plate and incubated at standard conditions for several days. At measure point cells were incubated with MTT solution (Roth, Karlsruhe, Germany) (5 mg/ml) for 4 h at 37°C, 5% CO_2_. Cells were then lysed with 100 μl Isopropanol + 0.1 N HCl and OD was measured with SLT spectra (Crailsheim, Germany) using Tecan X Fluor4 software.

### Image analysis

Cells were plated 2500/cm² in 12 well-plates on coverslips and cultured under standard condition for several days. Cells were than fixed with 4% PFA and stained against Actin (1:2000) overnight and DAPI (1:10000) for additional 5 min. Coverslips were mounted on slides with Immu-Mount (Thermo scientific, Massachusetts, USA). Pictures were taken with Olympus × 71 microscope with × 1000 magnitude. Exposure time was equal in different cell lines. Images were taken with cell-F software (Olympus, Tokyo, Japan).

### Cell cycle analysis

Cell cycle analysis was performed according to Pagliacci et al. [47]. Briefly, cells were seeded at a density of 8000 cells/cm² and held under standard conditions for 3 days. Cells were trypsinized and spun down, washed thrice in PBS and resuspended in 500 μl hypolysis buffer (0.1% sodium citrate, 0.1% Triton X-100, 100 g/ml RNAse A). Cells were then stained with propidiumiodid (50 μg/ml) and analyzed by fluorescence-activated cell sorting (FACS) as described. Cell cycle phases were set manually and analyzed with Cellsoft software.

### Sphenoid and migration assay

Cell migration assays were carried out as previously described [48–49]. In brief, cells were plated in 24 wells at 15,000 cells/cm² and held under standard conditions until confluence of 80%. Wound scratch was set using a 200 μl pipette. Floating cells were carefully removed. Plates were then held under standard conditions for additional 48 h. Pictures were taken with an Olympus IX71 microscope at different time points and analyzed with Image J software (NIH, USA) by measuring distance between the migrating cell boundaries as described [49]. For the sphenoid assay cells were embedded in methyl-cellulose and the invasion front was quantified as described [49].

### Colony forming assay

The colony-forming assay was adapted with modifications from Zhang et al. [50]. Briefly, cells were seeded (2000/cm²) in 1% soft agar/on top of a 2% soft agar layer. Cells were cultured under standard condition for 12 days. 4 pictures per well taken with Olympus ×71 and numbers of colonies were counted manually. Statistical analysis was performed with students *t*-test *p < 0.001 for wild type gliomas (WT) versus PRG3 and WT vs. PRG3^kd^.

### Data bank analysis

BioGPS databank analysis and mRNA expression value data for PRG3 measured by the Affymetrix U133A Chip were obtained from the online database BioGPS (http://biogps.gnf.org [51]. Graphics were processed using Microsoft Excel 2007. The web-based human cancer microarray database Oncomine (Compendia Biosciences; Ann Arbor, MI, USA; www.oncomine.org) was used to analyze the mRNA expression PRG3 in different malignant brain tissues. Details of the general standardized normalization techniques and statistical calculations can be found on the Oncomine website (https://www.oncomine.com). Briefly, we perform the normalization per dataset and per sample of the microarray data set instead of dataset-wide global normalization. For that we facilitated Freije Brain library at Oncomine. In total 59 samples of glioblastoma patients were measured via the U133A microarray. The measured data were processed as described at oncomine.org. For removing differences in dynamic range a log2 transformation was performed. To remove bias in signal intensity between samples data were then subtracted by the median PRG3 value from each individual value. The x-axis determines the median expression values for all genes measured by the U133A microarray. For our study we excluded samples from patients whose overall survival time was below 50 days or above 1000 days. PRG3 overexpression was defined as values more than 3 times higher than normalized value for each probeset/gene and reduced PRG3 levels (knockdown values) were defined as values below −1.5. Kaplan-Meier survival plots for glioblastoma patients with low and intermediate level of PRG3 mRNA expression were obtained from the Freije Brain library (ONCOMINE database) and processed with MS Excel.

### *In vivo* imaging

Animal experiments were done in congruence to the European Union guidelines for the use of laboratory animals according to Savaskan et al. The protocol for animal experimentation was approved by the Government of Middle Franconia (permission number 54.2531.31–8/06) Male Fisher rats weighing 180–200 g (Charles Rivers) were deeply anesthetized using intraperitoneal injection before fixing them in a stereotactic frame (David Kopf Instruments, Bilaney Consultants). Stably GFP, PRG3 and PRG3^kd^ expressing rat glioma cells were stereotactically implanted in a volume of 4 μl (8 × 10^4^) with a Hamilton syringe (VWR) into the right frontal lobe of the animals (2 mm lateral to bregma, depth 4 mm from dura). Tumor implantation was monitored 10 days after implantation using a clinical 3 Tesla MRI scanner (Siemens Healthcare, Siemens AG, Erlangen, Germany). A modified neurological deficit assessment was established with four clinical scales [39]. Rats were clinically checked every day and evaluated according to neurologically status (grade 0: normal; grade 1: tail weakness or tail paralysis; grade 2: hind leg paraparesis or hemiparesis; grade 3: hind leg paralysis or hemiparalysis; grade 4: complete paralysis (tetraplegia), moribund stage or death). Rats were sacrificed at grade 4. Grade 1 was defined to be the onset of neurological deficit.

### MRI and brain edema determination

MR imaging was performed on a clinical 3 Tesla MR scanner unit (Magnetom Tim Trio Human MRi system, Siemens Healthcare, Siemens AG, Erlangen, Germany) with a 40-mm-diameter, small field-of-view orbita surface coil as a receiver. Scout images and a 3DCISS sequence (repetition time = 9 ms, echo time = 5 ms, reconstructions with a slice thickness of 2 mm) were obtained in coronal, axial, and transverse planes to position the slices accurately. Ten coronal T1- and T2-weighted slices, each with 2 mm thickness and 0.2 mm separation (inter-slice gap) were then positioned on the transverse scout images to cover the tumor. T1-weighted images were acquired with a 384 × 307 matrix, field-of-view = 70 × 70 mm, repetition time = 507 ms, echo time = 17 ms, and a total scan time of 3 min 42 s. For contrast enhanced images, each animal received 1 ml/kg body weight of contrast agent (Gadovist, Bayer Pharma, Leverkusen, Germany) i.p. 10 min prior to the acquisition of T1-weighted sequences. The T2-weighted images were acquired with a 320 × 256 matrix, field-of-view = 91 × 91 mm, repetition time = 4500 ms, echo time = 158 ms, and a total scan time of 6 min 12 s. Imaging analysis was performed for each rat using Osirix (GNU General Public License build-in image processing software) to outline tumor volume on the T1-weighted contrast-enhanced images. Total tumor volume was calculated as the summed area on all slices, multiplied by the slice separation and compared to histology-derived tumor volume. Additionally, edema volume was measured by subtracting the tumor volumes as derived from T1 and T2-weighted images.

### Statistical analysis

Quantitative data from experiments were obtained as stated in the Figure legend. Analysis was performed using unpaired Student's *t* test if not otherwise stated (MS Excel). Data from all experiments were obtained from at least three independent experiments. For survival analysis we used the GraphPad Prism software (GraphPad Software, Inc., LaJolla, USA). The level of significance was set at *p < 0.05. Error bars represent ± SD if not otherwise stated.

## SUPPLEMENTARY MATERIALS FIGURES


