# Supplementary material for: PRG3 induces Ras-dependent oncogenic cooperation in gliomas
Source: Oncotarget. 2016 Apr 5;7(18):26692–708. doi: 10.18632/oncotarget.8592 (PMC5042008; doi:10.18632/oncotarget.8592)
Supplement: Supplementary file 1 [file oncotarget-07-26692-s001.pdf]

PRG3 induces Ras-dependent oncogenic cooperation in gliomas

Supplementary Materials

```
                 10         20         30         40         50         60
              TM1
hPRG-3   MAVGNNTQRS YSIIPCFIFV ELVIMAGTVL LAYYFECTDT FQVHIQGFFC QDGDLMKPYP
mPRG-3   MAVENNTQRS YSIIPCFIFV ELVIMAGTVL LAYYFECTDT FQVHIQGFFC QDGDLMKPYP
rPRG-3   MAVENNTQRS YSIIPCFIFV ELVIMAGTVL LAYYFECTDT FQVHIQGFFC QDGDLMKPYP

             TM2 70         80         90        100        110        120
hPRG-3   GTEEESFITP LVLYCVLAAT PTAIIFIGEI SMYFIKSTRE SLIAQEKTIL TGECCYLNPL
mPRG-3   GTEEESFISP LVLYCVLAAT PTAIIFIGEI SMYFIKSTRE SLIAEEKMIL TGDCCYLSPL
rPRG-3   GTEEESFISP LVLYCVLAAT PTAIIFIGEI SMYFIKSTRE SLIAEEKMIL TGDCCYLSPL

            TM3 130        140        150        160        170        180
hPRG-3   LRRIIRFTGV FAFGLFATDI FVNAGQVVTG HLTPYFLTVC KPNYTSADCQ AHHQFINNGN
mPRG-3   LRRIIRFIGV FAFGLFATDI FVNAGQVVTG HLTPYFLTVC QPNYTSTDCR AHQQFINNGN
rPRG-3   LRRIVRFIGV FAFGLFATDI FVNAGQVVTG HLTPYFLTVC QPNYTSTDCR AHHQFINNGN

                190        200 TM4    210        220        230 TM5    240
hPRG-3   ICTGDLEVIE KARRSFPSKH AALSIYSALY ATMYITSTIK TKSSRLAKPV LCLGTLCTAF
mPRG-3   ICTGDLEVIE KARRSFPSKH AALSIYSALY ATMYITSTIK TKSSRLAKPV LCLGTLCTAF
rPRG-3   ICTGDLEVIE KARRSFPSKH AALSIYSALY ATMYITSTIK TKSSRLAKPV LCLGDLCTAF

                250    TM6 260        270        280        290        300
hPRG-3   LTGLNRVSEY RNHCSDVIAG FILGTAVALF LGMCVVHNFK GTQGSPSKPK PEDPRGVPLM
mPRG-3   LTGLNRVSEY RNHCSDVIAG FILGTAVALF LGMCVVHNFR GTQGSPSKPK PEDPRGVPLM
rPRG-3   LTGLNRVSEY RNHCSDVIAG FILGTAVALF LGMCVVHNFK GTQGSASKPK PEDPRGVPLM

                310        320        330
hPRG-3   AFPRIESPLE TLSAQNHSAS MTEVT.....
mPRG-3   AFPRIESPLE TLSAQNHSAS MTEVT*....
rPRG-3   AFPRIESPLE TLSAQNHSAS MTEVT.....
```

Supplementary Figure S1: PRG3 amino acid sequence alignment of human, rat and mouse species. Amino acid sequence of human PRG3 (GenBank Accession no. AY337718) aligned to mouse PRG3 (GenBank Accession no. AY345342) and rat PRG3 (GenBank Accession no. AY299399). Putative transmembrane domains TM1 - TM6 are boxed (black lines).

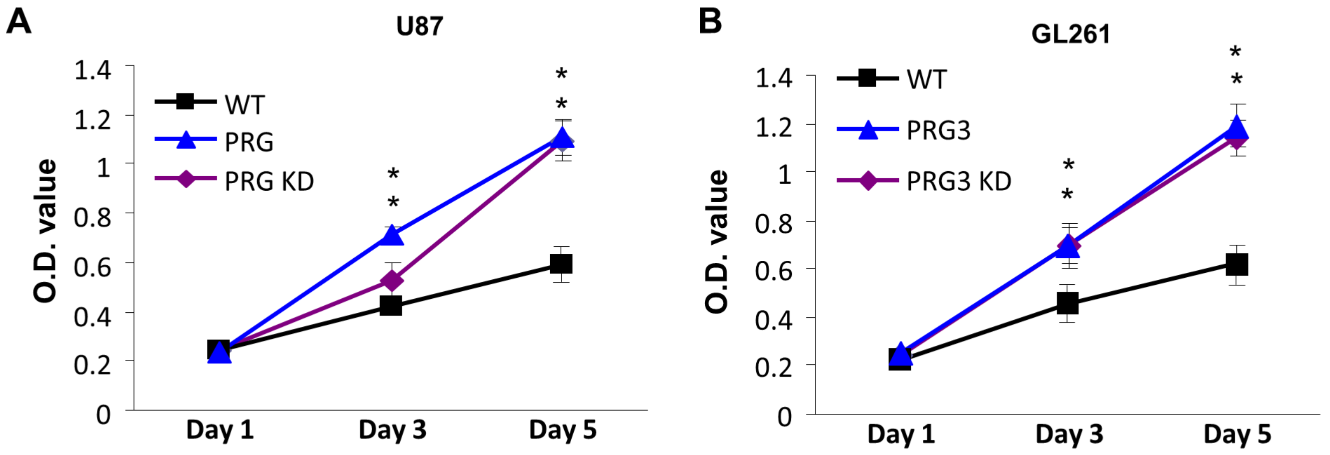

Supplementary Figure S2: PRG3 expression enhances human tumor proliferation. (**A**) PRG3 boosts growth in human glioma cells. Quantitative growth analysis of human glioma cells (U87) over time. Note that PRG3 overexpressing cells grow significantly faster than wild-type (WT) gliomas. Statistical significance was calculated with Student's *t*-test (mean ± SD, $n > 12$ per group; $^*P < 0.05$). (**B**) PRG3 boosts growth in murine glioma cells. Quantitative analysis of cell growth in murine glioma cells (GL-261) over time. Note that PRG3 overexpressing cells grow significantly faster than RFP control transfected cells. Statistical significance was calculated with Student's *t*-test (mean ± SD, $n = 12$ per group; $^*P < 0.05$). Abbreviation: OD, optical density.

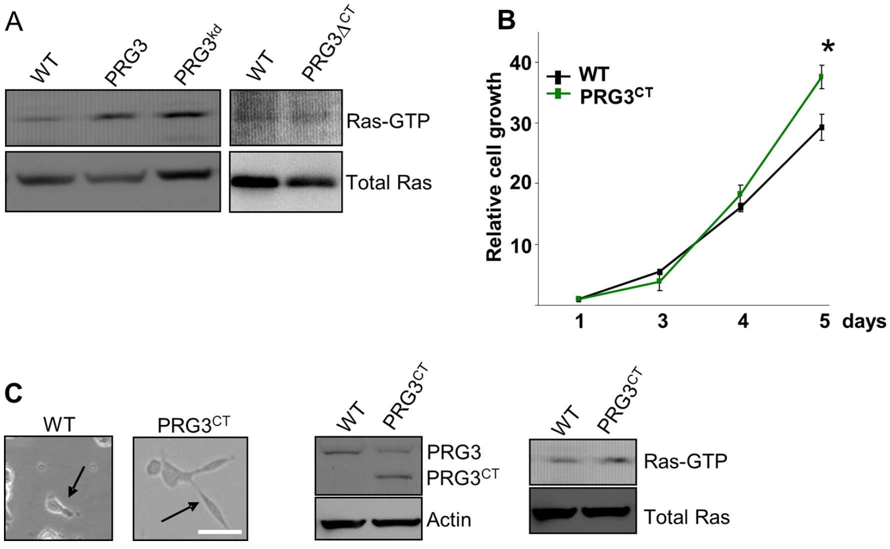

Supplementary Figure S3: (A) *Left*, **Perturbed PRG3 expression leads to elevated Ras activation.** Upper blot gives levels of activated GTP-Ras in wild-type (WT), PRG3 overexpressing (PRG3) and PRG3-knockdown ($PRG3^{kd}$) gliomas. Total Ras served as controls for equal loading (bottom panel). *Right*, C-terminal tail deleted PRG3 construct does not affect Ras signaling. (**B**) PRG3 promotes cell growth through its C-terminal domain. Quantitative analysis of cell growth over time of parental (WT) and PRG3-C-terminal domain expressing ($PRG3^{CT}$) glioma cells. Statistical significance was calculated with Student's *t*-test (mean ± SD, $n = 4$ per group; $^*P < 0.005$). (**C**) $PRG3^{CT}$ expression in gliomas alters cellular morphology and activates Ras. *Left*, representative images showed PRG3-C-terminal domain expressing ($PRG3^{CT}$) glioma cells exert an elongated cell shapes comparable to PRG3 full length expressing gliomas. Middle, Upper blot shows $PRG3^{CT}$ expression levels in wild-type (WT) and $PRG3^{CT}$ glioma cells. Actin served as a control for equal loading (bottom panel). *Right*, Upper panel shows activated Ras-GTP in wild-type (WT) and $PRG3^{CT}$ glioma cells. Total Ras served as a control for equal loading (bottom panel). Scale bar represents 50 μm.

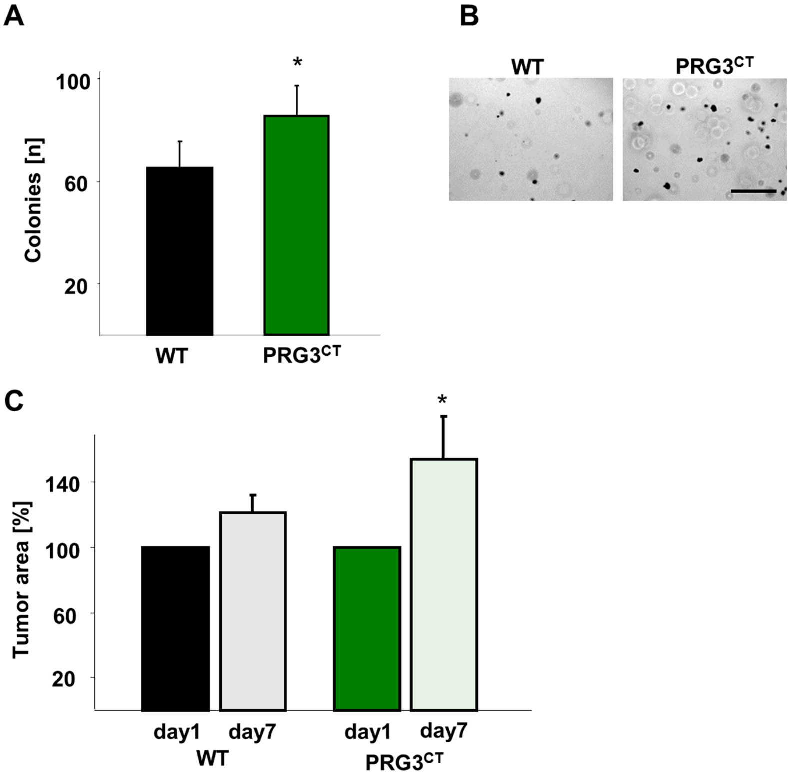

Supplementary Figure S4: The C-terminal domain of PRG3 promotes oncogenesis and glioma growth. Oncogenic amplification of glioma cells after expression of the C-terminal domain of PRG3. (**A**) Quantitative analysis of colony formation in parental wild-type glioma cells (WT) and PRG3 C-terminal domain expressing gliomas ($PRG3^{CT}$). Quantification is given from three independent experiments. Values are given as mean ± SD, *$P < 0.001$ (Student's unpaired *t*-test). (**B**) Representative images of colonies formed in soft agar from wild-type glioma cells (WT) and PRG3 C-terminus expressing gliomas ($PRG3^{CT}$). Scale bar, 500 µm. (**C**) Expression of PRG3 C-terminal domain in gliomas ($PRG3^{CT}$) fosters tumor expansion into brain parenchyma. GFP+ glioma cells (WT) and PRG3 C-terminal domain expressing gliomas ($PRG3^{CT}$) were implanted in brain slices and tumor expansion was evaluated after various days. Quantitative analysis of tumor expansion (bright colored columns represent tumor expansion at day 1; faintly colored columns represent tumor expansion at day 7). Statistical significance was calculated with Student's unpaired *t*-test (mean ± SD, $n = 6$ per group; *$P < 0.005$).
